# The Dengue virus in Nepal: gaps in diagnosis and surveillance

**DOI:** 10.1186/s12941-018-0284-7

**Published:** 2018-07-16

**Authors:** Birendra Prasad Gupta, Andrea Haselbeck, Jerome H. Kim, Florian Marks, Tarun Saluja

**Affiliations:** 1 Central Diagnostic Laboratory and Research Centre Pvt. Ltd, Kathmandu, Nepal; 2 International Vaccine Institute, Seoul, Republic of Korea; 3 Department of Medicine, Cambridge University, Cambridge, UK

**Keywords:** Epidemiology, Surveillance, Dengue, Nepal, Diagnosis

## Abstract

**Background:**

The introduction of the dengue virus (DENV) in Nepal is recent, first reports date back to 2004 from a Japanese traveller and limited information is available about DENV infection in the Nepali population. Within a decade after the first DENV detection, it is now endemic in multiple districts of Nepal with approximately 11.2 million people residing in the Terai belt being at risk of DENV infection. Sporadic cases of DENV infection have been reported every year for the past decade during the monsoon season, mainly in the Terai region.

**Methods:**

Medline/Embase/Cochrane databases were reviewed for reports on the burden of dengue infection, diagnostic methods, and national surveillance.

**Results:**

Four outbreaks were reported since 2004 including the diagnosis of all serotypes in 2006 and predominance of a single serotype in 2010 (DENV-1), 2013 (DENV-2), and 2016 (DENV-1). The clinical diagnoses showed a predominance of dengue fever while 4/917 (0.4%), 8/642 (1.2%) and 8/1615 (0.4%) dengue haemorrhagic fever/dengue shock syndrome cases were identified during the outbreaks in 2010, 2013 and 2016, respectively. The number of cases reported in males was significantly higher (67.4%) than in females. Disease occurrence was primarily found in the Terai region until 2010 and was increasingly detected in the Hilly region in 2016.

**Conclusion:**

In Nepal currently weak diagnostic facilities, very limited research on mosquitoes vectors, and poor surveillance of dengue leading to inappropriate detection and control of DENV. We surmise that improved basic research and epidemiological training courses for local scientists and laboratory personal at national and international level will help better understand the evolution and distribution of DENV transmission and its eventual control.

## Background

The first record of a clinically dengue like disease was recorded in the Chinese medical encyclopaedia from the Jin Dynasty (265–420 AD) [1]. It was referred to as “water poison” associated with flying insects at that time [2]. Civilization and human migration contributed to the spread of dengue and its primary vectors, *Aedes aegypti* and *Aedes albopictus*, to new geographical areas [3]. A large epidemic of dengue was seen in different cities of Japan during WWII, including Nagasaki, Kobe, and Osaka [4] with 200,000 dengue cases in the years 1942–1948 [5]. It seems the dengue virus infections originated from travellers who were returning from Southeast Asia and the Pacific islands [6]. Dengue was first recognized in Manila, the Philippines in 1953 [7]. Viruses similar to DENV-1 and DENV-2 were isolated from Manila patients by William Hammond and were called DENV-3 and DENV-4 in 1956 [8]. Similarly, Dengue viruses with multiple serotypes were isolated from patients of a dengue haemorrhagic fever (DHF) outbreak in Bangkok, Thailand in 1958 [9], and dengue outbreaks were mainly reported from Southeast Asia till the early 1980s [10].

Similarly, epidemiology of dengue in India is extremely complex and changing. The first reported occurrence of dengue fever dated to 1946, and no outbreaks were reported for almost 20 years until an epidemic occurred in Kolkata in 1963–1964 [11]. In Delhi, the first major outbreak of dengue fever/dengue haemorrhagic fever (DF/DHF) occurred in Delhi in 1996, after three decades of very low incidence, with 10,252 cases and 423 deaths [12]. India reported 28,292 cases and 110 deaths in 2010 which is the highest number of cases and number deaths in a single year in the country in the previous two decades [13]. Co‐circulation of multiple dengue virus serotypes and has been seen in Uttar Pradesh and Kerala, India during the last decade [14, 15].

Nepal is a landlocked Himalayan country bordering India in the East, West, and South and China’s autonomous Tibet region in the North. The population of Nepal is 26,494,504 (2011 census) [16]. The country is divided into three ecological regions (Terai, Hilly and Mountain Regions) and 75 districts. The population is unevenly distributed in these three ecological regions. The population is unevenly distributed, with 48.4% of the Nepalese populous residing in the Terai region and the remaining in the Hill and Mountain regions. Each district has one district hospital (government public hospital), and numerous private/non-governmental hospitals, nursing homes and medical colleges. The public hospitals commonly have their own laboratory for routine diagnosis which lacks a molecular laboratory for the confirmation of pathogens. The other private hospitals rarely have molecular diagnosis and if so, it is very costly.

Although the Nepalese culture and daily lifestyle largely resembles that of India, dengue fever was not diagnosed for > 50 years while India already routinely reported cases. Here, we provide an overview of the epidemiology of dengue fever in three different Nepalese regions and suggest local laboratory infrastructure improvements, particularly for the Terai region to ensure appropriate diagnosis and surveillance.

## Methods

We searched the databases Medline/Embase/Cochrane for scientific literature published in the past 10 years for relevant evidence on the burden of dengue infection, diagnostic methods, and surveillance strategies/activities and discussed current gaps in diagnosis and surveillance in Nepal. The following search terms were used “dengue”, “diagnosis”, “epidemiology”, “Nepal”.

### Dengue virus detection and epidemic pattern in Nepal

Sparse information on DENV infection in Nepal was available prior to 2004 [17], which changed with the report of one Japanese foreigner being diagnosed with dengue fever in 2004 [18]. Since then, four DENV outbreaks occurred in Nepal, with the first endogenous dengue outbreak in the Chitwan district dating to 2006 [19]. At that time, the circulation of all four DENV serotypes was found in nine districts of the low-land Terai region [20]. Although the circulation of all four serotypes was reported during 2006–2007 dengue outbreaks, dengue serotype-1 (DENV-1) strains, closely related to Indian strains, were exclusively identified in the second outbreak in 2010 [21]. Similarly, only dengue serotype 2 (DENV-2) was identified during the third outbreak in 2013 [22, 23]. The Epidemiology and Disease Control Division (EDCD) from the Nepalese Ministry of Health documented 1615 dengue cases in 32 districts (out of 75 districts) during the fourth outbreak in 2016, predominantly DENV-1, with the disease even reaching into the highlands (Table 1) where most of the cases reported were dengue fever while 4/917 (0.4%), 8/642 (1.2%) and 8/1615 (0.4%) DHF/DSS cases were reported during 2010, 2013 and 2016 outbreak, respectively [24]. Similarly, the number of cases reported in males (n = 2248) was significantly higher than in females (n = 1086) i.e. 67.4% [24]. This might be because males have a higher vector exposure due to their predominance in outdoor work, especially in fields and forests. Overall, the majority [1836/3334 (55.1%)] of the dengue infections occurred in adults aged 15–40 years which may be associated with the lack of immunity in this age group living in areas where dengue was recently reported [22]. The proportion of laboratory confirmed cases (n = 3634) among clinically diagnosed cases (10,966) is about 30% [25]. Dengue outbreaks were mainly diagnosed during the monsoon and post-monsoon periods, with a peak from August to October.

**Table 1:** Circulation of dengue serotypes in Nepal

Years	No. of sample tested	DENV serotype	Percentage of dominant serotype	Epidemic/non-epidemic	References
2004	32	2	100%	Non-epidemic	Takasaki et al. [18]
2006	276	1, 2, 3, 4	25%	Non-epidemic	Malla et al. [26]
2007	100	Not studied	NA	Non-epidemic	Dumre et al. [28]
2008	25	Not Studied	NA	Non-epidemic	Dumre et al. [28]
2009	35	Not studied	NA	Non-epidemic	Dumre et al. [28]
2010	1215	1	100%	Epidemic	Pandey et al. [23]
2013	2340	2	100%	Epidemic	Gupta et al. [22]
2014	576	2	100%	Non-epidemic	Acharya et al. [20]
2015	545	Not studied	NA	Non-epidemic	Gupta et al. [24]
2016	4125	1	100%	Epidemic	Gupta et al. [25]

**Fig. 1:**
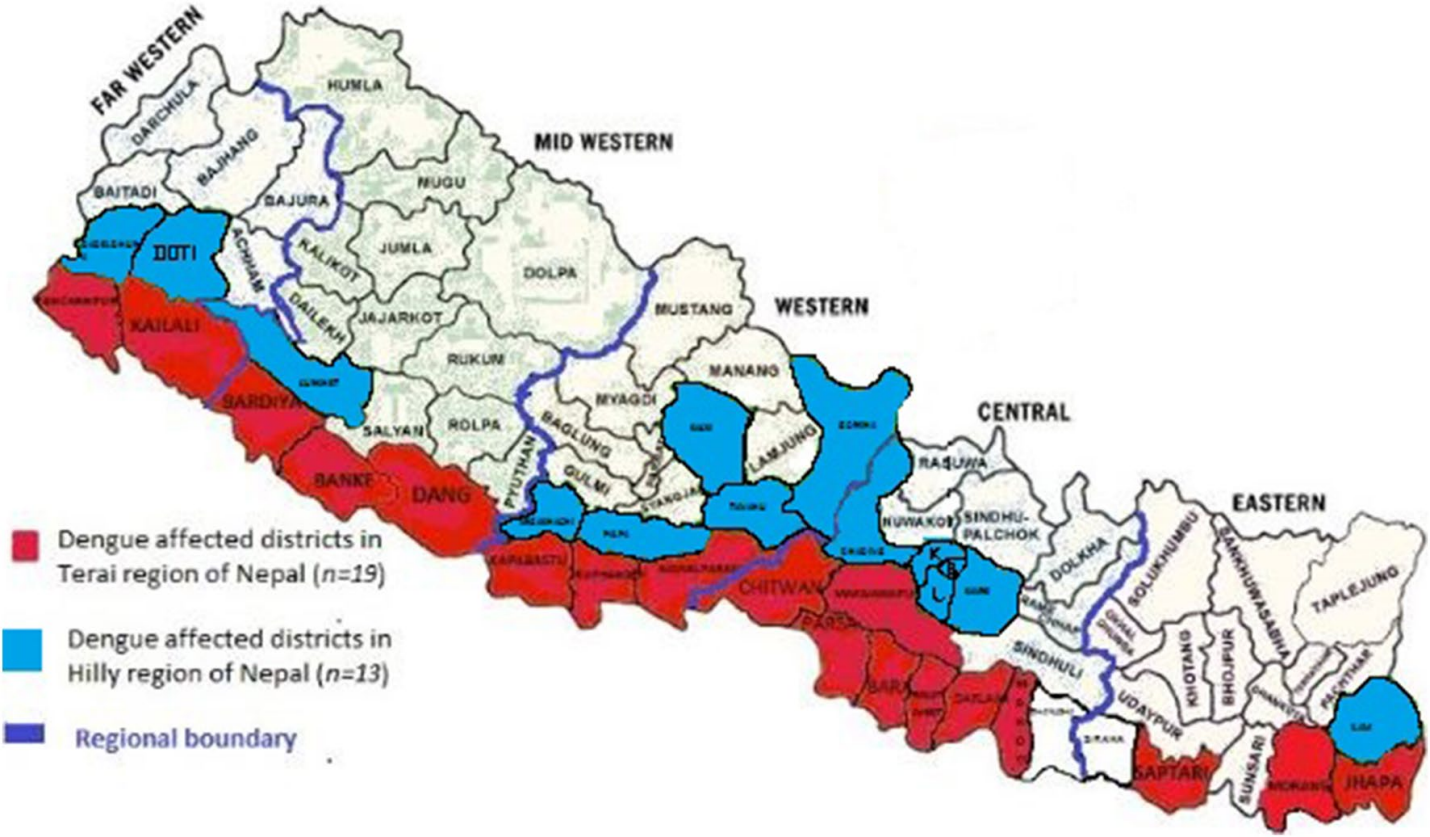
Distribution of dengue cases from 2006 to 2016 in Nepal

Geographically, dengue was first reported in Hilly region in 2010, and there are no reports of dengue cases from Mountain region published as of today (Fig. 1). There is an increasing trend of dengue in the Terai region potentially becoming a major public health concern, where it occurred at sporadic and/or epidemic rates with approximately 11.2 million residents at risk of DENV infection. Currently, due to the lacking laboratory equipment in government hospitals RDT kits (SD Bioline Dengue Duo) are only provided during the occurrence of an outbreak and are missing for dengue diagnostic on a regular basis despite the increasing disease burden during the last decade (Table 2).

**Table 2:** Health care facilities and dengue affected districts in Nepal

Region	Districts	Number of districts hospital	Number of molecular diagnosis laboratory in government hospitals	Dengue reported districts	Number of dengue case
Terai	20	20	NA	19	1473
Hilly	39	39	NA	13	46
Mountain	16	16	NA	Nil	Nil

## Discussion

DENV infection is endemic in Nepal with outbreaks every 3 years since the first endogenous dengue detection in Nepal [23]. All four serotypes are circulating as sporadic cases throughout the year; however, a specific single serotype was found predominantly responsible for outbreaks that occurred after 2010 in the past [26]. A serotype-shift occurred over the years with a predominance of DENV-1in 2010, DENV-2 in 2013 and again DENV-1 in 2016 [25].

The dengue burden in Nepal is exacerbated by the open border between Nepal and India, poor availability of medical and diagnostic facilities; inadequate mosquito control and the climatic conditions that favour vector expansion in the Nepal. DENV infection in Nepal is more common in the Terai region compared to the Hilly and Himalayan region presumably due to its ideal vector environment and the porous border between India and Nepal [27]. The vector has adapted to the extremes of warm and cold weather resulting in the occurrence of dengue cases throughout the year in Nepal.

In the past separate virus laboratories for the diagnosis of viral diseases were lacking in Nepal. Nowadays, laboratories that provide reliable confirmation of dengue infection are few and are based in Kathmandu, the capital city of Nepal. However, the majority of the population and specifically those at risk have no access to these facilities. Recently diagnosis by commercial kits is available to many hospitals through the EDCD, Ministry of Health, and Government of Nepal. We strongly suggest improving and facilitating the diagnostic capacity for proper diagnosis and surveillance through well-equipped centres in the Terai region. This will contribute to identifying “hidden” viral pathogens as early as possible and, hence, may prevent the emergence of outbreaks. The Government of Nepal, specifically the Ministry of Health, should prioritize appropriate staffing, equipping and training of medical facilities, including medical personnel at the primary health centre level and enable them to perform initial DENV diagnosis.

## Conclusion

Efforts are needed in order to develop improved, proactive, laboratory-based surveillance systems that can forecast impending dengue epidemics in this country. This will help alert the public to take action and physicians to diagnose and properly treat DF/DHF/DSS cases in the Terai region. Strong coordination between the Ministry of Health, Government of Nepal, EDCD, Nepal Public Health Laboratory (NPHL) and local hospital laboratories is necessary for dengue detection, confirmation and management. We do not think that current efforts are adequate for the diagnosis and management of dengue. Current capacities are limited to the stage of crisis management with rapid diagnostic kits. The implementation of systematic dengue surveillance including continuous laboratory supply for reliable diagnosis is needed to identify the real disease burden and to understand the geographical disease distribution.

## Abbreviations

DF: dengue fever
DHF: dengue haemorrhagic fever
DSS: dengue shock syndrome
DENV-1: dengue virus serotype-1
DENV-2: dengue virus serotype-2
DENV-3: dengue virus serotype-3
DOHs: Department of Health Services
EDCD: Epidemiology and Disease Control Division
ELISA: enzyme linked immunosorbent assay
IVI: International vaccine institute
NA: not available
ND: not done
NPHL: Nepal Public Health Laboratory
PCR: polymerase chain reaction
RDT: rapid diagnostic kit
